# A Disease-Associated Mutation Impedes PPIA through Allosteric Dynamics Modulation

**DOI:** 10.1021/acs.biochem.5c00260

**Published:** 2025-06-30

**Authors:** Yoshikazu Hattori, Munehiro Kumashiro, Hiroyuki Kumeta, Taisei Kyo, Soichiro Kawagoe, Motonori Matsusaki, Tomohide Saio

**Affiliations:** † Institute of Advanced Medical Sciences, 13109 Tokushima University, Tokushima 770-8503, Japan; ‡ Faculty of Advanced Life Science, 12810 Hokkaido University, Sapporo 001-0021, Japan; § Student Laboratory, Faculty of Medicine, Tokushima University, Tokushima 770-8503, Japan

## Abstract

Amyotrophic lateral sclerosis (ALS) is a progressive neurodegenerative disease characterized by motor neuron degeneration. Peptidylprolyl *cis–trans* isomerase A (PPIA) is a molecular chaperone involved in protein folding, and its dysfunction has been linked to ALS pathogenesis, as proline is recognized as a key residue for maintaining proper folding of ALS-related proteins. A recent study identified a K76E mutation in PPIA in sporadic ALS patients, but its effect on protein function and structure remain unclear. In this study, we used biochemical and biophysical techniques to investigate the structural and functional consequences of the K76E mutation. Our results show that K76E significantly reduces enzyme activity without affecting structure, monodispersity, or substrate recognition. Significant effects of K76E mutation were identified by relaxation dispersion NMR experiments, showing that K76E disrupts key protein dynamics and alters an allosteric network essential for isomerase activity. Corroborated by theoretical kinetic analysis, these dynamics data, revealing the exchange process for K76E to be approximately 1 order of magnitude slower than that of the wild type, explain the reduced *cis–trans* isomerase activity of the K76E mutant. These findings suggest that the pathogenic effect of K76E arises primarily from impaired protein dynamics rather than direct structural disruption. Our study provides new insights into the molecular mechanisms underlying ALS-associated mutations and their impact on protein function.

## Graphical abstract

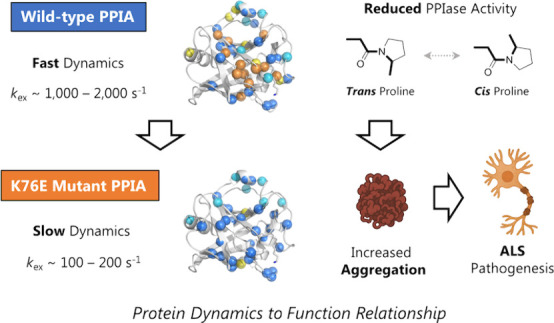

Amyotrophic lateral sclerosis (ALS) is a fatal neurodegenerative disease caused by motor neuron degeneration, which displays cytoplasmic inclusions.^1,2^ In the processes of ALS pathogenesis, proteins such as transactive response DNA-binding protein 43 (TDP-43), fused in sarcoma, and superoxide dismutase 1 (SOD1) misfold and aggregate, leading to neuronal damage.^3,4^ Moreover, molecular chaperones have gained attention as ALS-associated factors due to their roles in preventing protein misfolding and aggregation.^5,6^ Among them, peptidylprolyl *cis–trans* isomerase A (PPIA, also known as Cyclophilin A), a molecular chaperone possessing peptidylprolyl *cis–trans* isomerase (PPIase) activity,^7,8^ has been proposed as an ALS-related protein.^9^ Indeed, increased levels of TDP-43 and mutant SOD1 aggregates were observed in the spinal cord of PPIA knockout mice, which exhibited accelerated disease onset and progression.^10^ Another study showed that PPIA influenced the polymer structure of heterogeneous nuclear ribonucleoprotein A2 (hnRNPA2), another protein linked to ALS.^11^ Furthermore, proline mutations in TDP-43 and hnRNPA2 caused aggregation by stabilizing the self-associated structure.^12^ Thus, PPIA activity is connected to the molecular mechanism of ALS pathogenesis.

More recently, the K76E mutation in PPIA was identified in patients with sporadic ALS.^13^ Although the Lys residue can be an acetylation site and a previous study indeed showed that acetylation at K125 in PPIA favored interaction with TDP-43,^10^ to our knowledge, K76 has not been identified as an acetylation site (Figure S1). It was reported that the mutant is less stable and degraded more rapidly in cells than the wild-type protein.^13^ Furthermore, molecular dynamics (MD) simulations suggested that K76E mutation induces local structural changes around residues 26–30 and 43–45, which are possibly related to the destabilization of K76E.^13^ However, molecular insights are limited, and the mechanism of PPIA malfunction caused by K76E mutation remains to be elucidated.

This study focuses on the PPIase activity of PPIA K76E, with biochemical and biophysical measurements. Our biochemical data show that the K76E mutation reduces PPIase activity. Despite the distant location of K76 from the substrate-binding site, our biophysical data, particularly from nuclear magnetic resonance (NMR) relaxation measurements, highlight suppression of global conformational dynamics on the ms-μs time scale by the K76E mutation. Given the importance of the global conformational dynamics for PPIase activity, the suppression explains the allosteric effect of K76E on PPIase activity.

We first focused on the impact of the K76E mutation on PPIase activity and performed a spectroscopic assay using RNase T1 refolding where the slow prolyl *cis–trans* isomerization is the rate-limiting step.^14,15^ Figure 1A shows the refolding profiles of RNase T1 in the absence and presence of wild-type PPIA and the K76E mutant. In the presence of wild-type PPIA, refolding was strongly accelerated, reflecting its PPIase activity. Refolding was also accelerated in the presence of K76E; however, the acceleration was less pronounced than that observed with wild-type PPIA. This small but significant difference between wild-type PPIA and K76E revealed that the K76E mutation in PPIA reduces the PPIase activity (Figure 1B).

**Figure 1:**
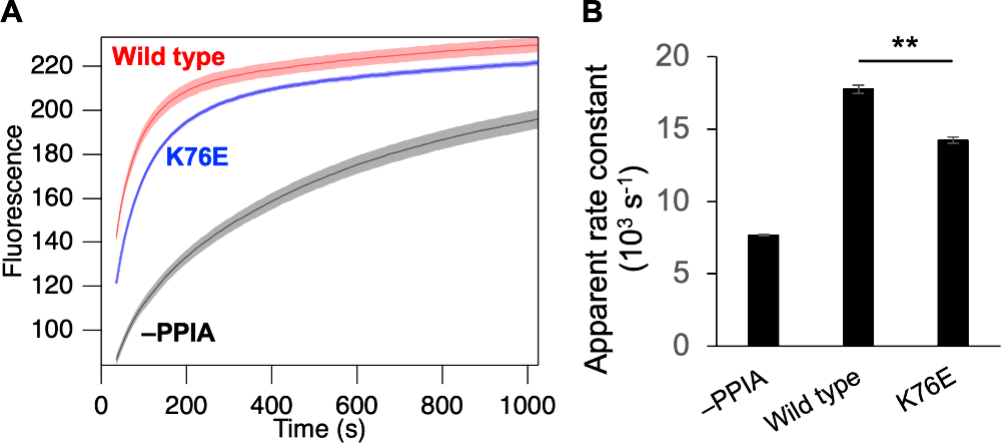
(A) PPIase activity assessed by monitoring the refolding of RNase T1. Fluorescence increases during RNase T1 refolding in the absence of PPIA (black) and in the presence of wild-type PPIA (red) or K76E (blue). Shaded areas represent the standard deviation (*N* = 3). (B) Apparent rate constants derived from fitting the refolding curves (up to 200 s) to a single exponential function. Statistical significance between wild-type PPIA and K76E was assessed using Welch’s *t*-test. ** indicates *p* < 0.01.

Our data also showed that the K76E mutation alters PPIase activity without significantly affecting its substrate binding affinity. NMR titration experiments using the model peptides Suc-AAPF-pNA,^7^ which has been commonly used as a substrate in PPIase activity assays, and Ac-FGPDLPAGD-NH_2_,^16^ which has been shown to exhibit higher affinity to PPIA, revealed that the K76E mutation caused negligible change in the affinity (Table 1 and Figures S2 and S3). The decreased PPIase activity of K76E might potentially be caused by protein aggregation. To investigate aggregation properties, size-exclusion chromatography coupled with multiangle light scattering (SEC-MALS) analysis was performed (Figure S4). The SEC profiles of wild-type PPIA and K76E both showed sharp peaks with similar elution times. The molecular sizes determined by MALS were 18.2 ± 0.4 kDa for wild-type PPIA and 18.3 ± 0.4 kDa for K76E, consistent with theoretical value for monomeric PPIA (18.5 kDa for wild-type PPIA and K76E). These data showed that K76E exists as a monodisperse monomer in solution; therefore, the decrease in PPIase activity of K76E is not attributable to differences in aggregation.

**Table 1:** Dissociation Constants between Wild-Type PPIA or K76E and Substrates^a^

	Suc-AAPF-pNA	Ac-FGPDLPAGD-NH_2_
Wild type (mM)	4.4 ± 0.3	0.32 ± 0.1
K76E (mM)	3.7 ± 0.2	0.36 ± 0.1

a Determined by NMR titration experiments from fitting chemical shift perturbations to the standard quadratic binding equation for a 1:1 interaction. Errors represent fitting uncertainty.

The decrease in catalytic activity of the K76E mutant might also be attributed to structural changes within the catalytic center (around active residue R55). To assess this, chemical shift perturbation (CSP) was analyzed using ^1^H–^15^N correlation NMR spectra measured for wild-type PPIA and the K76E mutant (Figure 2). Overlaying the K76E spectrum onto the wild-type spectrum revealed changes in only a limited number of peaks. CSP mapping showed that most perturbed resonances belong to residues structurally adjacent to the mutation site. The region near the catalytic center was not significantly perturbed. Thus, the K76E mutant appeared to retain a structure of the catalytic center similar to that of the wild type. Accordingly, we concluded that the reduced activity is unlikely due to structural changes in the catalytic center caused by the mutation.

**Figure 2:**
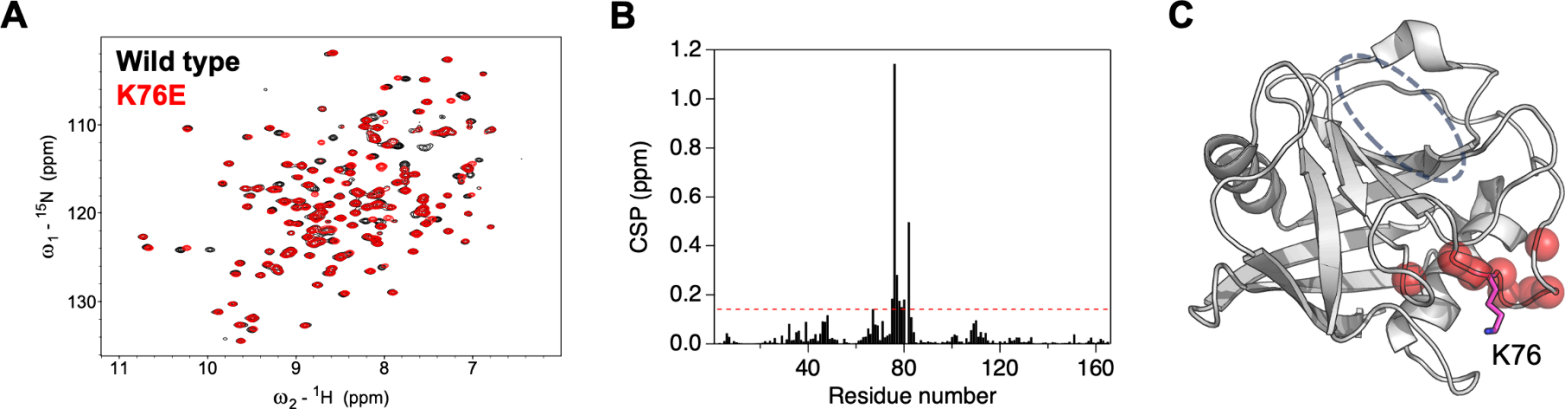
(A) Overlay of ^1^H–^15^N correlation spectra of wild-type PPIA (black) and K76E (red). (B) Chemical shift perturbation (CSP) calculated from ^1^H–^15^N correlation spectra of wild-type PPIA and K76E. Red dashed line indicates the threshold value (mean +1 SD). (C) Residues exhibiting CSP above the threshold are mapped as red spheres onto the structure of PPIA (PDB ID: 1RMH). The K76 side chain is shown as a magenta stick. The putative substrate binding site is indicated by a gray dashed line.

We also investigated the dynamics of PPIA, given a previous report showing PPIA possesses intrinsic conformational dynamics in a range of 1,000 to 2,000 s^–1^ that are essential for PPIase activity.^17^ To assess the impact of K76E mutation on the global conformational dynamics of PPIA, Carr–Purcell–Meiboom–Gill (CPMG) relaxation dispersion NMR was performed on wild-type PPIA and K76E. For the wild type, several residues located around the substrate-binding site and near K76 showed significant dispersion, indicating relatively fast exchanges processed with rates of a few thousand s^–1^ (Figures 3A,B and S5, and Table S1). As proposed in previous studies,^17–19^ these global conformational dynamics drive the *cis–trans* isomerization of the substrate captured by PPIA. In contrast, the relaxation dispersion profiles for residues in PPIA K76E showed an obvious change; exchange rates of only a few hundred s^–1^ were observed (Figure 3A,B and S5, and Table S1). These results suggest that the enzymatically important exchange processes are attenuated by the K76E mutation. Indeed, prediction of the allosteric network using Ohm^20^ revealed that K76 is part of an allosteric network connected to the catalytic center of PPIA (Figure S6). It should be noted that allosteric pathways include the regions where Pasetto et al. suggested local structural changes,^13^ providing a potential link between the studies. Consistent with the previous data showing the reduced stability of PPIA K76E in the cell,^13^ our data from circular dichroism (CD) measurements showed the melting temperature (*T*_m_) of PPIA K76E is approximately 5 °C lower than that of the wild type (Figure S7). This implies that the mutation induces a change in the population between the two states. Thus, the data indicate that the K76E mutation disrupts the allosteric network of PPIA and consequently impedes its functional dynamics.

**Figure 3:**
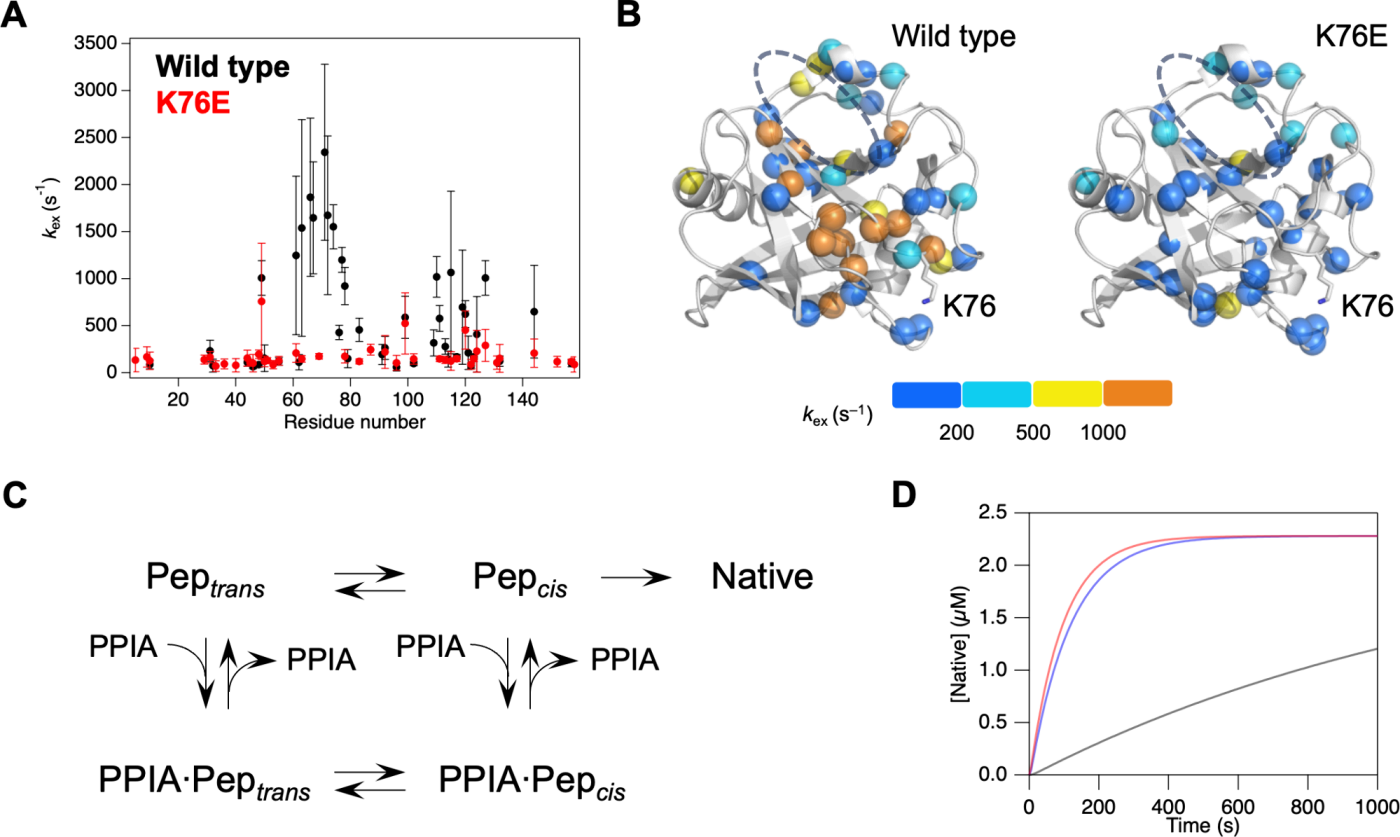
(A) Exchange rate constants (*k*_ex_) for wild-type PPIA (black) and K76E (red) obtained from fitting ^15^N CPMG relaxation dispersion data to the Luz-Meiboom equation for the fast exchange regime. Error bars represent fitting errors. Some of the resonances in K76E mutant showed severe line broadening and thus are omitted in the CPMG analysis. (B) Exchange rate constants (*k*_ex_) mapped onto the structure of PPIA (PDB ID: 1RMH). Residues are shown as spheres colored according to the *k*_ex_ value (stepwise color scale shown below). The putative substrate binding site is indicated by a gray dashed line. (C) Five-state kinetic model used for simulations of RNase T1 refolding catalyzed by PPIA. (D) Simulated RNase T1 refolding curves in the absence of PPIA (black), presence of wild-type PPIA (red), and presence of K76E (blue), based on the kinetic model in (C) and parameters in Table S1.

Given the significant reduction in conformational dynamics, PPIA K76E was expected to exhibit markedly reduced *cis–trans* isomerization activity toward its substrate. This hypothesis was supported by a five-state kinetic model (Figure 3C) reconstituting RNase T1 refolding. Since the refolding processes of RNase T1 are rather complicated, we chose simple model and kinetic parameters following the previous studies (Table S2).^18,21^ The refolding curves were reasonably simulated if the rate of *cis–trans* isomerization on PPIA K76E was set to approximately one-tenth of that for the wild type (Figures 3D, S8, and S9). This coincides with the approximate difference in CPMG-derived *k*_ex_ values between wild-type PPIA and K76E, and therefore indicates that the *cis–trans* isomerization on PPIA K76E was reduced to approximately one-tenth that of the wild type. Therefore, the small but significant reduction in the RNase T1 refolding rate observed for the K76E mutant was explained by a drastic reduction of PPIase activity.

Collectively, our biochemical and biophysical data show that the K76E mutation reduces the enzymatic activity of PPIA through the modulation of the dynamics on the ms-μs time scale. Previous studies reported the concerted global dynamics of PPIA on the ms-μs time scale both in the absence and presence of the substrate and proposed that the dynamics is coupled with *cis–trans* isomerization of the substrate.^17–19,22^ Our study unveiled that a mutation at K76 disrupts the conformational dynamics and PPIase activity of PPIA. Interestingly, K76 is located in one of the two allosteric networks in PPIA, as reported in the previous study,^19^ and the catalytic residue R55 is included in the same network as K76, suggesting that the K76E mutation disrupts the concerted dynamics in the allosteric network (Figure S6). Our data regarding dynamics on the ms-μs scale complement previous results from MD simulations on the order of a few μs.^13^ Although modulations of local fluctuations of PPIA by K76E mutation in ns time scale were probed by MD simulations especially for the loop regions (residues 26–30 and 43–45), the impact of the dynamics modulations on enzyme activity was not understood. In this study, we focused on dynamics on a slower time scale and highlighted a significant effect of K76E on the dynamics on the ms-μs scale that is crucial to the enzymatic activity of PPIA. This allosteric dynamics modulation attenuates the enzymatic activity of PPIA, resulting in a disruption of protein homeostasis, which may have a cumulative effect on the ALS pathogenesis.

## Supplementary Material



## Notes

Additional experimental details, materials, methods, figures, and tables (PDF)

## Notes

UniProt ID for PPIA (P62937).

## References

[1] Rowland L. P., Shneider N. A. (2001). Amyotrophic lateral sclerosis. N. Engl. J. Med..

[2] Kiernan M. C., Vucic S., Cheah B. C., Turner M. R., Eisen A., Hardiman O., Burrell J. R., Zoing M. C. (2011). Amyotrophic
lateral
sclerosis. Lancet.

[3] Ling S.-C., Polymenidou M., Cleveland D. W. (2013). Converging mechanisms in ALS and
FTD: disrupted RNA and protein homeostasis. Neuron.

[4] Jucker M., Walker L. (2013). Self-propagation of pathogenic protein aggregates in
neurodegenerative diseases. Nature.

[5] Muchowski P. J., Wacker J. L. (2005). Modulation of neurodegeneration
by molecular chaperones. Nat. Rev. Neurosci..

[6] Ciechanover A., Kwon Y. T. (2017). Protein quality
control by molecular chaperones in
neurodegeneration. Front. Neurosci..

[7] Fischer G., Wittmann-Liebold B., Lang K., Kiefhaber T., Schmid F. X. (1989). Cyclophilin and
peptidyl-prolyl cis-trans isomerase
are probably identical proteins. Nature.

[8] Takahashi N., Hayano T., Suzuki M. (1989). Peptidyl-prolyl
cis-trans isomerase
is the cyclosporin A-binding protein cyclophilin. Nature.

[9] Nigro P., Pompilio G., Capogrossi M. C. (2013). Cyclophilin
A: a key player for human
disease. Cell Death Dis.

[10] Lauranzano E., Pozzi S., Pasetto L., Stucchi R., Massignan T., Paolella K., Mombrini M., Nardo G., Lunetta C., Corbo M., Mora G., Bendotti C., Bonetto V. (2015). Peptidylprolyl
isomerase A governs TARDBP function and assembly in heterogeneous
nuclear ribonucleoprotein complexes. Brain.

[11] Xiang S., Kato M., Wu L. C., Lin Y., Ding M., Zhang Y., Yu Y., McKnight S. L. (2015). The LC
domain of
hnRNPA2 adopts similar conformations in hydrogel polymers, liquid-like
droplets, and nuclei. Cell.

[12] Zhou X., Sumrow L., Tashiro K., Sutherland L., Liu D., Qin T., Kato M., Liszczak G., McKnight S. L. (2022). Mutations
linked to neurological disease enhance self-association of low-complexity
protein sequences. Science.

[13] Pasetto L., Grassano M., Pozzi S., Luotti S., Sammali E., Migazzi A., Basso M., Spagnolli G., Biasini E., Micotti E., Cerovic M., Carli M., Forloni G., De Marco G., Manera U., Moglia C., Mora G., Traynor B. J., Chiò A., Calvo A., Bonetto V. (2021). Defective cyclophilin A induces TDP-43
proteinopathy: implications for amyotrophic lateral sclerosis and
frontotemporal dementia. Brain.

[14] Babu M., Favretto F., de Opakua A. I., Rankovic M., Becker S., Zweckstetter M. (2021). Proline/arginine
dipeptide repeat polymers derail protein
folding in amyotrophic lateral sclerosis. Nat.
Commun..

[15] Kawagoe S., Nakagawa H., Kumeta H., Ishimori K., Saio T. (2018). Structural
insight into proline cis/trans isomerization of unfolded proteins
catalyzed by the trigger factor chaperone. J.
Biol. Chem..

[16] Piotukh K., Gu W., Kofler M., Labudde D., Helms V., Freund C. (2005). Cyclophilin
A binds to linear peptide motifs containing a consensus that is present
in many human proteins. J. Biol. Chem..

[17] Eisenmesser E. Z., Millet O., Labeikovsky W., Korzhnev D. M., Wolf-Watz M., Bosco D. A., Skalicky J. J., Kay L. E., Kern D. (2005). Intrinsic
dynamics of an enzyme underlies catalysis. Nature.

[18] Holliday M. J., Armstrong G. S., Eisenmesser E. Z. (2015). Determination of the full catalytic
cycle among multiple cyclophilin family members and limitations on
the application of CPMG-RD in reversible catalytic systems. Biochemistry.

[19] Holliday M. J., Camilloni C., Armstrong G. S., Vendruscolo M., Eisenmesser E. Z. (2017). Networks of dynamic allostery regulate
enzyme function. Structure.

[20] Wang J., Jain A., McDonald L. R., Gambogi C., Lee A. L., Dokholyan N. V. (2020). Mapping
allosteric communications within individual
proteins. Nat. Commun..

[21] Schiene C., Reimer U., Schutkowski M., Fischer G. (1998). Mapping the stereospecificity
of peptidyl prolyl cis/trans isomerases. FEBS
Lett..

[22] Eisenmesser E. Z., Bosco D. A., Akke M., Kern D. (2002). Enzyme dynamics during
catalysis. Science.

