# Supplementary material for: A Disease-Associated Mutation Impedes PPIA through Allosteric Dynamics Modulation
Source: Biochemistry. 2025 Jun 30;64(14):2971–5. doi: 10.1021/acs.biochem.5c00260 (PMC12269060; doi:10.1021/acs.biochem.5c00260)
Supplement: Supplementary file 1 [file bi5c00260_si_001.pdf]

Supporting Information

A Disease-Associated Mutation Impedes PPIA through Allosteric Dynamics Modulation

Yoshikazu Hattori [a], Munehiro Kumashiro [a], Hiroyuki Kumeta [b], Taisei Kyo [a,c], Soichiro Kawagoe [a], Motonori Matsusaki [a], and Tomohide Saio [a*]

[a] Institute of Advanced Medical Sciences, Tokushima University, Tokushima 770-8503, Japan

[b] Faculty of Advanced Life Science, Hokkaido University, Sapporo, 001-0021, Japan

[c] Student Laboratory, Faculty of Medicine, Tokushima University, Tokushima 770-8503, Japan

[*] Email: saio@tokushima-u.ac.jp

Materials and Methods

Protein Expression and Purification

Human wild-type PPIA (UniProt ID: P62937) and K76E fused with a His_6-GB1 tag and a TEV cleavage site at the N-terminus were overexpressed in the *E. coli* BL21(DE3) strain. The cells were grown at 37 ºC in LB medium or M9 minimal medium containing 1 g/L $^{15}NH_4Cl$ for unlabeled or ^{15}N-labeled samples, respectively. Protein expression was induced with 0.5 mM IPTG at an OD_{600} of 0.6-0.8, followed by cultivation at 15 ºC overnight. The cells were harvested and resuspended in lysis buffer containing 50 mM Tris, pH 8.0, and 500 mM NaCl. The suspended cells were lysed using an ultrasonic homogenizer and centrifuged at 18,000 rpm for 30 min. The protein in the supernatant was purified using Ni-NTA agarose (QIAGEN), and TEV protease was added to the eluate during dialysis against 50 mM Tris, pH 8.0, and 150 mM NaCl. After digestion, the solution was purified again using Ni-NTA agarose to remove the His_6-GB1 tag and any uncleaved protein. The flow-through was further purified by size-exclusion chromatography using a HiLoad 26/600 Superdex 200 pg column (Cytiva) equilibrated with 20 mM potassium phosphate, pH 7.0, 100 mM KCl, 4 mM 2-mercaptoethanol, and 0.05% NaN_3.

Peptide Preparation

Suc-AAPF-pNA was purchased from Sigma-Aldrich. Ac-FGPDLPAGD-NH_2 was obtained from custom peptide synthesis service supplied by GenScript. Product purity was 92.6%.

RNase T1 Refolding Assay

RNase T1 refolding assay was performed as described previously.[1] Briefly, RNase T1 (Sigma-Aldrich) was denatured by incubating overnight with 6.9 M urea. Urea-denatured RNase T1 (12 μL) was rapidly diluted to 408 μL of pre-incubated buffer or PPIA solution (100 mM Tris, pH 8.0; dilution rate: 35-fold) to initiate refolding of RNase T1. The dead time was 20 s. The time-course of tryptophan fluorescence of 2.28 μM RNase T1 in the presence and absence of 0.05 μM PPIA (wild type and K76E) was monitored using a spectrofluorometer (FP-8350; JASCO). The excitation and emission wavelengths were set to 280 nm (bandwidth: 2.5 nm) and 320 nm (bandwidth: 10 nm), respectively. Each profile represents the average of three replicates recorded at a scan speed of 10 s^{-1} and response time of 8 s. The standard error was estimated from the three replicates. All the measurements were performed at 10 °C. The baseline (buffer- or PPIA-only control) was subtracted. The photobleaching was suppressed by placing a 1% dimmer over the excitation light window. To determine the apparent rate constant, the initial phase of the refolding curve (up to 200 s) was fitted to a single exponential function. This method focuses on the initial enzymatic rate and avoids complications from the multiphasic nature of the full RNase T1 refolding process.

Statistical Analysis

Statistical analysis was performed using Welch's *t*-test to compare initial rate constants between wild-type PPIA and K76E. Analysis was conducted in R (version 4.4.1). A p-value less than 0.05 was considered statistically significant. Sample sizes for each group are indicated in the figure legend.

SEC-MALS

SEC-MALS was measured using DAWN HELEOS8+ (Wyatt Technology Corporation, Santa Barbara, CA, USA), a high-performance liquid chromatography pump LC-20AD (Shimadzu, Kyoto, Japan), refractive index detector RID-20A (Shimadzu), and UV-vis detector SPD-20A (Shimadzu), located downstream of the Shimadzu liquid chromatography system and connected to a PROTEIN KW-803 gel filtration column (Shodex). Differential RI (Shimadzu) downstream of MALS was used to determine protein concentrations. The running buffer comprised 20 mM potassium phosphate, pH 7.0, 100 mM KCl, 4 mM 2-mercaptoethanol, and 0.05% NaN_3. The 100 µL of 50 µM wild-type PPIA or K76E was injected at a flow rate of 1.0 mL min^{-1}. The data were analyzed using ASTRA version 7.0.1 (Wyatt Technology Corporation). Molar mass analysis was performed over half the width of the top height of the UV peak.

NMR

For the measurements of ^{1}H-^{15}N correlation spectra of wild-type PPIA and K76E, the samples were dissolved in 20 mM potassium phosphate, pH 7.0, 100 mM KCl, 4 mM 2-mercaptoethanol, and 0.05% NaN_3 containing 5% D_2O at a protein concentration of 0.1 mM. The measurements were performed on a Bruker AVANCE III 500 MHz spectrometer equipped with a BBFO probe at 25 ºC. Backbone resonance assignments of wild-type PPIA were obtained from BMRB entry 27265. The resonances of K76E were assigned mainly by tracing the resonances of the wild type and were additionally confirmed by using triple resonance data, including HNCA, HNCOCA, HNCACB, and CBCACONH, which were acquired with a Bruker AVANCE III HD 600 MHz spectrometer equipped with a TBI probe at 25 ºC. The data were processed using NMRPipe.[2] The spectra were analyzed using POKY.[3] The weighted average of the chemical shift perturbation (CSP) was calculated using following equation:

$$\mathrm{CSP} = \sqrt{\Delta\delta_{\mathrm{H}}^{2} + \left(\frac{\Delta\delta_{\mathrm{N}}}{5}\right)^{2}} \tag{1}$$

where $\Delta\delta_H$ and $\Delta\delta_N$ are the ^{1}H and ^{15}N chemical shift changes, respectively.

For the titration experiments with substrates, the samples were dissolved in 50 mM sodium phosphate, pH 6.5, and 1 mM dithiothreitol. NMR spectra were measured on a Bruker AVANCE III 500 MHz spectrometer equipped with a BBFO probe at 25 ºC. The titration with Suc-AAPF-pNA was performed at a 0.5 mM protein concentration. A 200 mM stock solution of Suc-AAPF-pNA in dimethylsulfoxide-d_6 was added to the protein solution to achieve final molar ratios of 2, 4, 8, and 16. Samples were prepared separately to maintain a final concentration of 4% dimethylsulfoxide-d_6. The titration with Ac-FGPDLPAGD-NH_2 was performed at a 0.2 mM protein concentration. A 20 mM stock solution of Ac-FGPDLPAGD-NH_2 in buffer was added sequentially to the protein solution to achieve final molar ratios of 0.5, 1, 2, 4, and 8. The data were processed using NMRPipe.[2] The spectra were analyzed using POKY.[3] Dissociation constants (K_d) were determined by fitting the observed chemical shift changes (Δ_{obs}) to the standard quadratic binding equation for a 1:1 interaction:[4]

$$\Delta_{\mathrm{obs}} = \frac{\Delta_{\mathrm{max}}}{2}\left[\frac{1 + X + K_{\mathrm{d}}}{[\mathrm{P}]_0} - \left\{\left(\frac{1 + X + K_{\mathrm{d}}}{[\mathrm{P}]_0}\right)^{2} - 4X\right\}^{½}\right] \tag{2}$$

where X is the molar ratio of substrate to protein, $[P]_0$ is the initial concentration of protein, Δ_{max} is the chemical shift changes between the fully bound and unbound forms, and Δ_{obs} is the chemical shift changes between at a given titration point relative to the unbound form. In the case of the titration with Suc-AAPF-pNA, ^{1}H chemical shift changes from five residues were globally fitted with the equation because ^{1}H shift changes were dominant. In the case of the titration with Ac-FGPDLPAGD-NH_2, the weighted averaged chemical shift changes (calculated using the CSP equation above) for 10 residues were globally fitted using the binding equation.

For the measurements of ^{15}N TROSY-based CPMG relaxation dispersion,[5] wild-type PPIA and K76E were prepared at a 0.7 mM concentration in 50 mM sodium phosphate, pH 6.5, and 1 mM dithiothreitol. NMR spectra were measured on a Bruker AVANCE Neo 800 MHz spectrometer equipped with a cryogenic TCI probe at 10 ºC. CPMG pulses were applied at frequencies (ν_{CPMG}) of 31.25, 62.5, 125, 250, 375, 500, 625, 750, and 1000 Hz during a 16 ms mixing period. The data were processed using NMRPipe.[2] Two-site exchange rate constants (k_{ex}) were extracted using GLOVE[6] by the fitting the data to the Luz-Meiboom equation:[7]

$$R_{2,\mathrm{eff}} = R_{2,0} + \frac{p_\mathrm{A} p_\mathrm{B} \Delta\omega^2}{k_\mathrm{ex}}\left(1 - \frac{4\nu_\mathrm{CPMG}}{k_\mathrm{ex}}\tanh\left(\frac{k_\mathrm{ex}}{4\nu_\mathrm{CPMG}}\right)\right) \quad (3)$$

where $R_{2,\mathrm{eff}}$ is the effective transverse relaxation rate, $R_{2,0}$ is the transverse relaxation rate in the absence of chemical exchange, p_A and p_B are the populations of the states in the two-state model, $\Delta\omega$ is the chemical shift difference between the states, and ν_{CPMG} is the frequency of the CPMG pulses.

Mapping Allosteric Communications

Allosteric coupling intensities within PPIA residues were predicted for the atomic coordinate of PDB ID: 1RMH using Ohm web-server.[8] An active site was designated to R55. The distance cutoff is set to 3.4 Å, and the parameter 'alpha' is set to 3.

CD Spectroscopy for Thermal Stability Analysis

The CD spectra of PPIA were recorded in the temperature range of 25-70 °C using a commercial CD spectrometer (J-1500, Jasco) and a 1 mm path-length cuvette. The CD spectrum at each temperature was measured three times and averaged. The CD spectrum of the buffer solution (100 mM Tris-HCl (pH 8)) was also measured as a background and subtracted from the spectra of PPIA. All of the CD spectra were collected from 260 to 200 nm (band width: 1 nm) with a 4 s response time, a 20 nm min^{-1} scan speed. The midpoint temperature, T_m, of the thermal unfolding of PPIA was estimated by fitting the temperature dependence of CD at 223 nm, $I(T)$, with a sigmoid function:

$$I(T) = I_\mathrm{u} + m_\mathrm{u}(T - T_\mathrm{u}) + \frac{I_n - I_\mathrm{u} + m_\mathrm{n}(T - T_\mathrm{n}) - m_\mathrm{u}(T - T_\mathrm{u})}{1 + \exp\left(-\frac{\Delta H}{RT}\left(1 - \frac{T}{T_\mathrm{m}}\right)\right)} \quad (4)$$

where T is the absolute temperature, T_n and T_u are the reference temperatures for PPIA in the native (25 °C) and the unfolded states (70 °C), respectively; I_n and I_u are the CD at 25 °C and 70 °C, respectively; ΔH is the molar enthalpy change of the transition at T_m; and R is the gas constant. m_n and m_u in the equation are temperature dependences of the CD of PPIA in the native and unfolded states, which are empirical parameters. The adjustable parameters in the

equation are ΔH, T_m, m_n, and m_u.

Theoretical Kinetic Analysis

Protein folding in which proline *cis-trans* isomerization is the rate-limiting step was simulated in the presence and absence of PPIA (wild type and K76E) using Kintek Explorer (v.11.1.1; Kintek Corporation).[9] The mechanism tested here was built upon the known mechanism for minimal reaction model of PPIA isomerization,[10] which consisted of four states and eight reaction pathways, as shown in Figure 3C. The microscopic rate constants were set based on previous studies, as listed in Table S2. Based on the NMR results, the rate constants for the transition between PPIA:Pep_{trans} and PPIA:Pep_{cis} for PPIA K76E were fixed to approximately one-tenth of those for the wild type. To account for the folding pathway after isomerization, a pathway from Pep_{cis} to Native states was included in the model. The rate constant for the Pep_{cis}-Native transition was set to 0.1 s^{-1}, which roughly reproduces the refolding behavior of RNase T1 in the presence and absence of PPIA (Figure 3D, S8, and S9). Differential equation of the kinetic model was numerically solved under the initial conditions of 2.28 μM Pep_{trans}, 0 or 0.05 μM PPIA, and 0 μM other components.

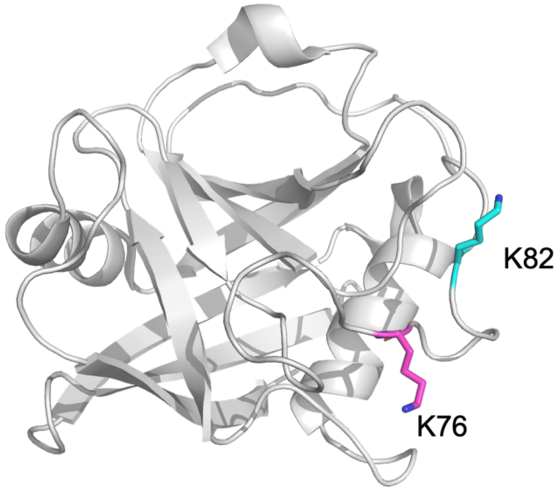

Figure S1. The position of key lysine residues on the structure of PPIA (PDB ID: 1RMH). K82, which has been reported as an acetylation site, is shown as a stick model. K76 is shown as a magenta stick model.

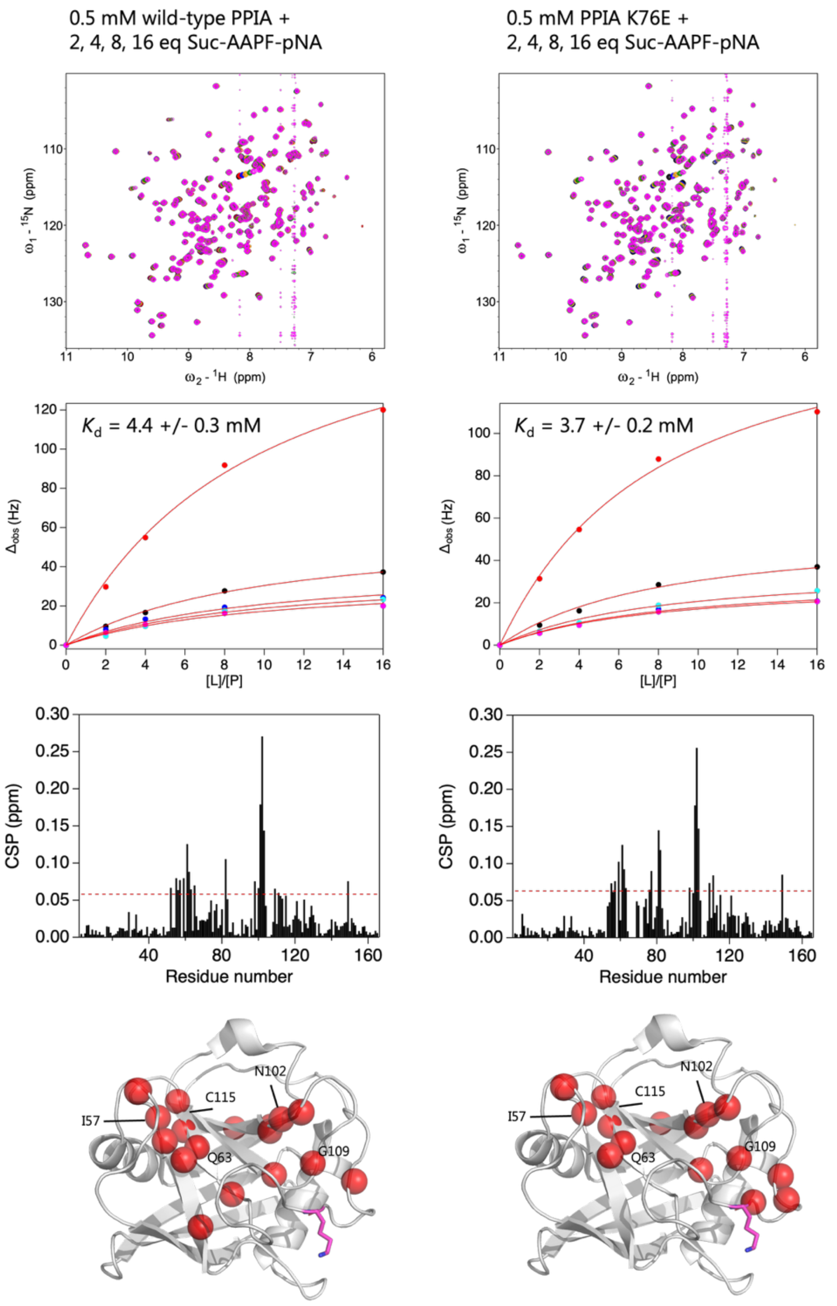

Figure S2. NMR titration experiments of wild-type PPIA (left column) and K76E (right column) with Suc-AAPF-pNA. Spectra in the absence (black) and presence of 2 (blue), 4 (orange), 8 (green), and 16 (magenta) molar equivalents of Suc-AAPF-pNA are overlaid. Titration curves for the five residues (I57, Q63, N102, G109, and C115) used for global fitting with the equation (2) are shown. Bar graphs for chemical shift perturbation (CSP) are shown with red dashed lines indicating the threshold value (mean + SD). Residues exhibiting CSP above the threshold are mapped as red spheres onto the structure of PPIA (PDB ID: 1RMH). The K76 side chain is shown as a magenta stick. The positions of residues that used for fitting are listed.

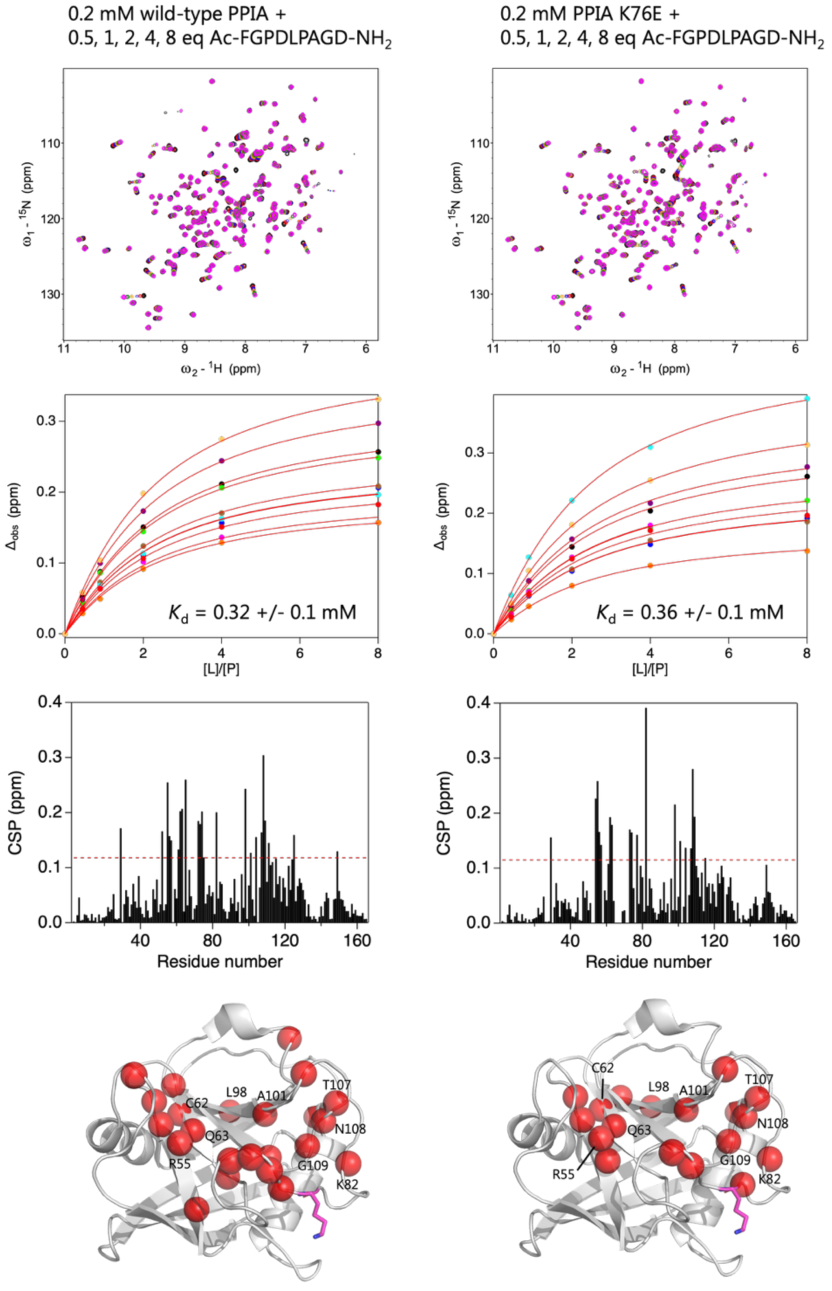

Figure S3. NMR titration experiments of wild-type PPIA (left column) and K76E (right column) with Ac-FGPDLPAGD-NH_2. Spectra in the absence (black) and presence of 0.5 (red), 1 (blue), 2 (orange), 4 (green), and 8 (magenta) molar equivalents of Ac-FGPDLPAGD-NH_2 are overlaid. Titration curves for the 10 residues (R55, C62, Q63, K82, L98, A101, T107, N108, G109, and W121 side-chain) used for global fitting with the equation (2) are shown. Bar graphs for chemical shift perturbation (CSP) are shown with red dashed lines indicating the threshold value (mean + SD). Residues exhibiting CSP above the threshold are mapped as red spheres onto the structure of PPIA (PDB ID: 1RMH). The K76 side chain is shown as a magenta stick. The positions of residues that used for fitting are listed.

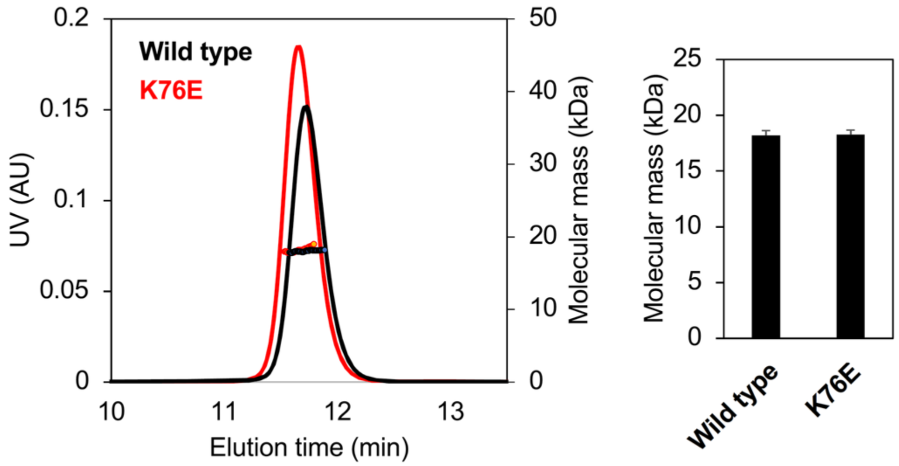

Figure S4. SEC-MALS experiments for wild-type PPIA and K76E. (Left) SEC profiles for wild-type PPIA (black) and K76E (red). (Right) Molecular masses determined by MALS. Theoretical molecular mass is 18.5 kDa for wild-type PPIA and K76E. Error bars represent standard deviations (N = 3).

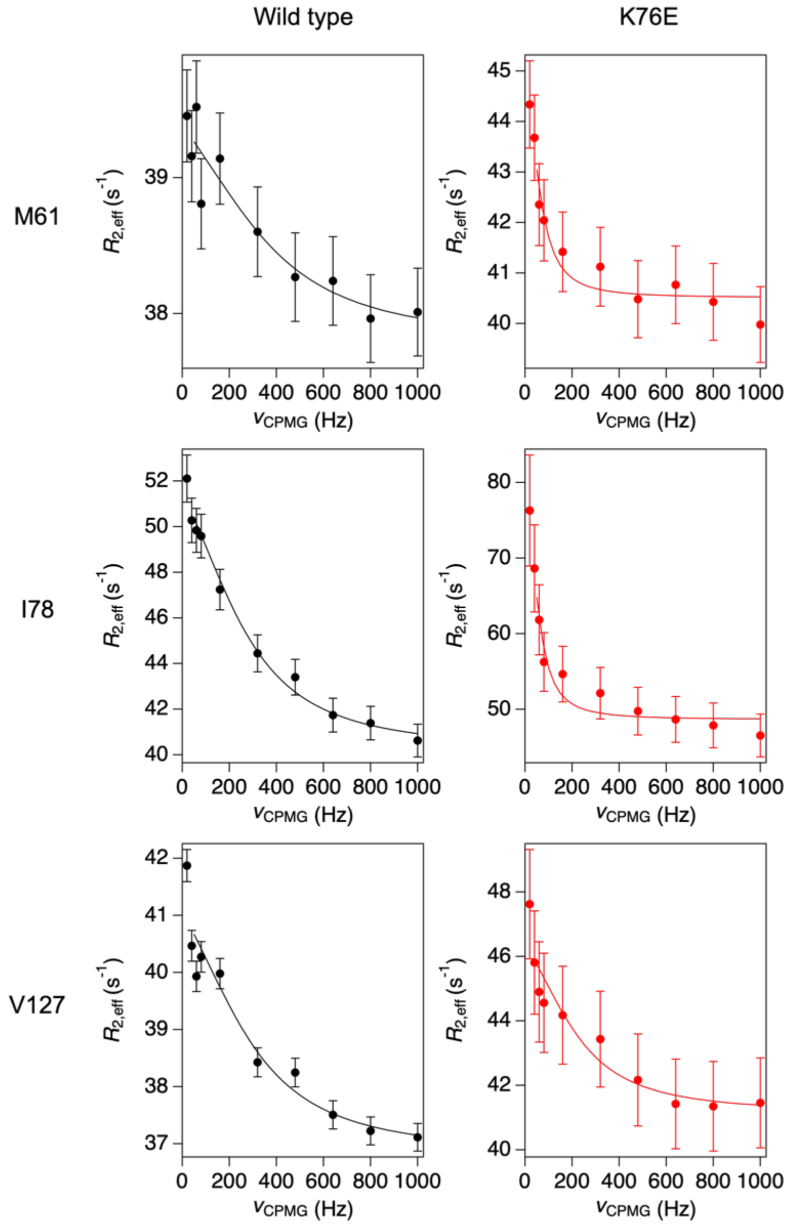

Figure S5. Representative ^{15}N CPMG relaxation dispersion profiles for M61, I78 and V127. The dots and solid lines are experimental and fitted values, respectively. Error bars were estimated from signal-to-noise ratios.

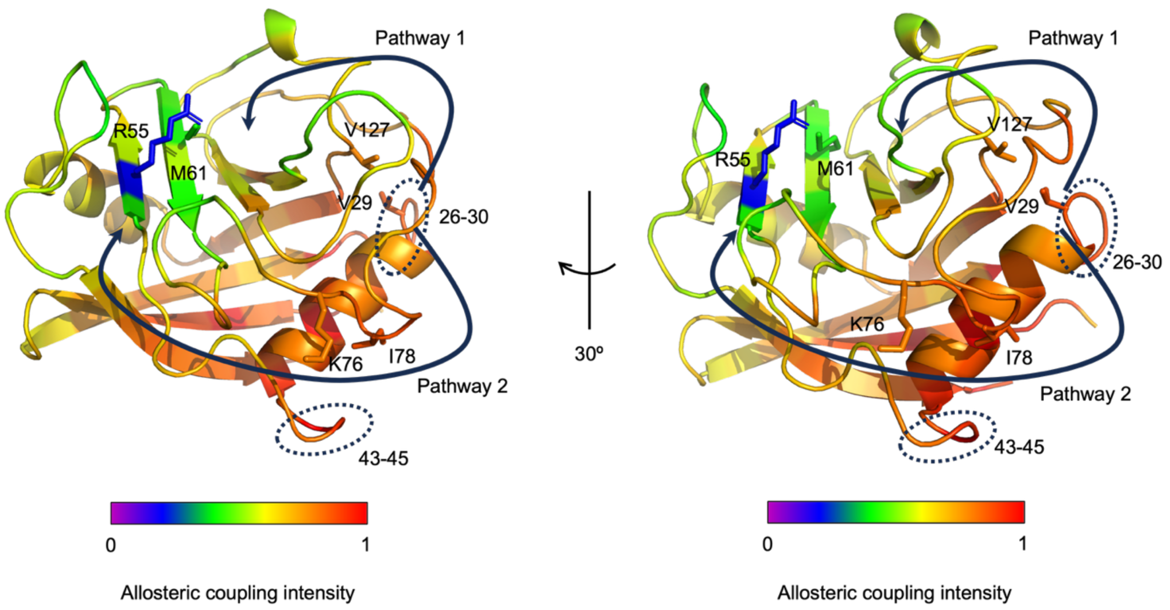

Figure S6. Allosteric coupling intensities from active site R55 calculated using Ohm are colored according to a rainbow gradient on the structure of PPIA (PDB ID: 1RMH). The scale bar is shown below. Higher value (red color) of allosteric coupling intensity means more allosteric coupling to the active site. Two allosteric pathways from the hotspot residue V29 supposed by Holliday et al.[11] are drawn by arrows. The positions of M61, I78, and V127, for which CPMG profiles are presented, are indicated.

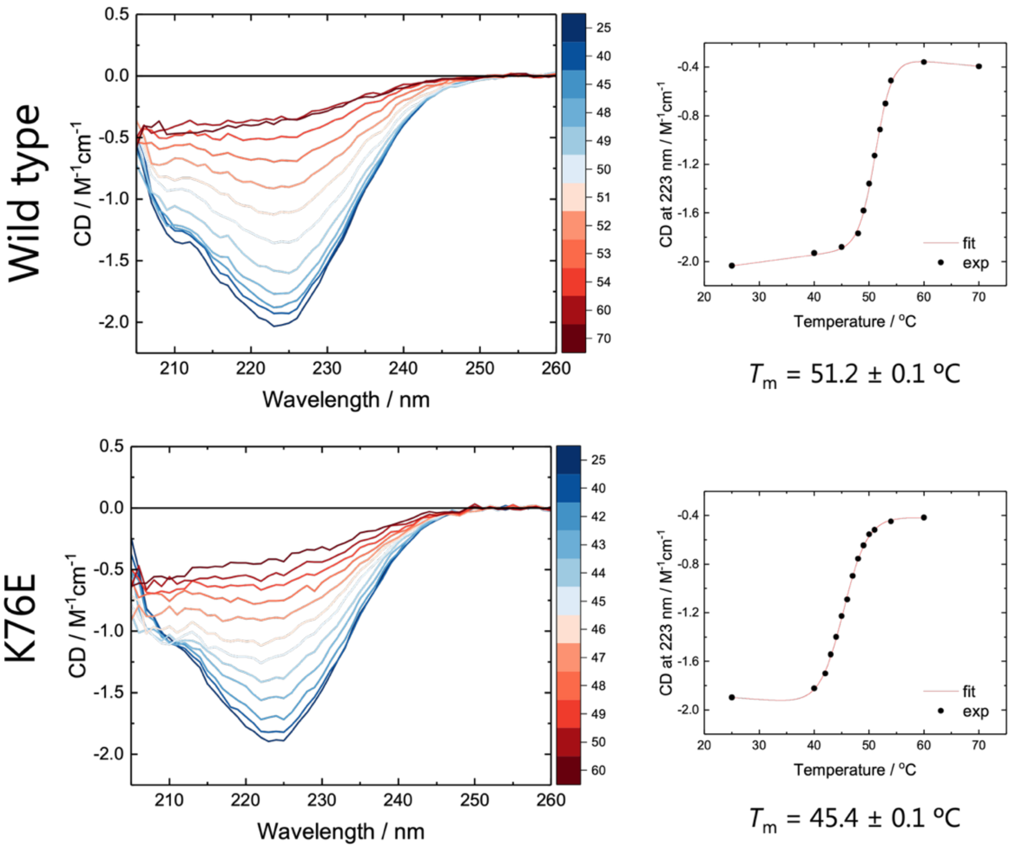

Figure S7. Thermal denaturation of wild-type PPIA and K76E monitored by circular dichroism (CD).

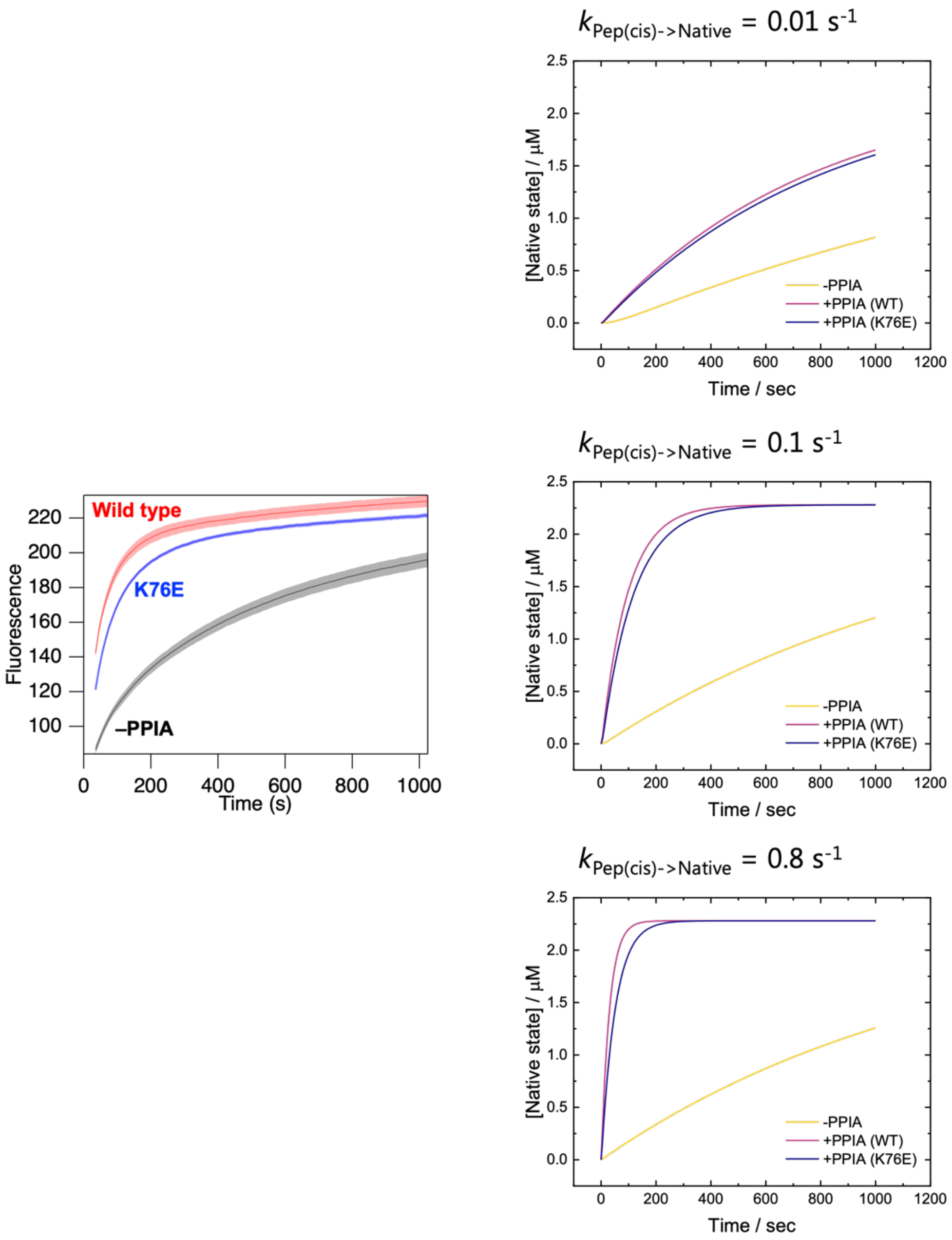

Figure S8. Comparison of experimental refolding curves (left) and theoretical refolding curves (right) by changing rate constants for the transition from the Pep_{cis} to the Native state. The experimental refolding curves on the left panel are the same as those shown in the Figure 1A for comparison.

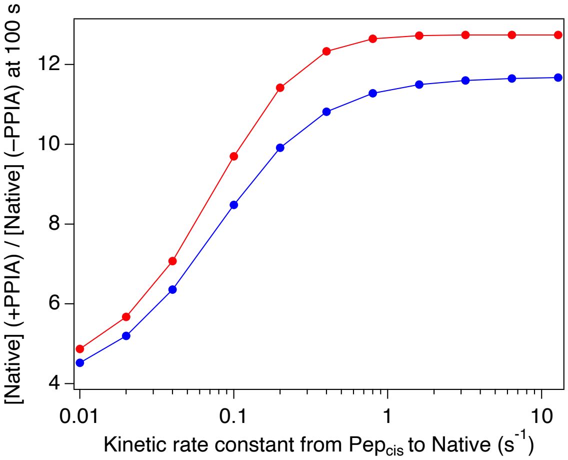

Figure S9. Optimization of rate constants for the transition from the Pep$_{cis}$ to the Native state in the kinetic model for wild-type PPIA (red) and K76E (blue).

Table S1. Exchange Rate constants obtained from CPMG relaxation dispersion analysis.

Residue	k_{ex} (wild type)	Error	k_{ex} (K76E)	Error
T5			1.3E+02	1.2E+02
D9			1.7E+02	1.1E+02
I10	8.6E+01	4.6E+01	1.3E+02	9.2E+01
V29			1.4E+02	4.7E+01
K31	2.3E+02	1.1E+02	1.6E+02	6.0E+01
T32	7.5E+01	6.8E+01		
A33			6.9E+01	5.7E+01
F36			9.3E+01	5.2E+01
S40			7.8E+01	7.1E+01
K44	1.1E+02	4.2E+01	1.5E+02	8.5E+01
G45			1.1E+02	3.3E+01
F46	6.3E+01	2.2E+01	1.0E+02	9.3E+01
Y48	8.6E+01	2.7E+01	1.9E+02	4.4E+01
K49	1.0E+03	1.8E+02	7.6E+02	6.2E+02
G50	1.5E+02	1.4E+02	1.2E+02	5.7E+01
S51			1.3E+02	3.0E+01
F53			8.6E+01	4.4E+01
R55	1.2E+02	4.0E+01	1.3E+02	4.6E+01
M61	1.2E+03	8.4E+02	2.1E+02	9.9E+01
C62	1.1E+02	8.1E+01		
Q63	1.5E+03	1.2E+03	1.5E+02	3.6E+01
D66	1.9E+03	8.4E+02		
F67	1.6E+03	6.0E+02		
R69			1.7E+02	2.9E+01
N71	2.3E+03	9.4E+02		
G72	1.7E+03	8.4E+02		
G74	1.6E+03	2.3E+02		
G75	4.3E+02	7.6E+01		
S77	1.2E+03	1.3E+02		
I78	9.2E+02	2.0E+02	1.7E+02	7.1E+01
Y79	1.5E+02	9.7E+01		
F83	4.6E+02	1.2E+02	1.2E+02	2.7E+01
N87			2.4E+02	5.7E+01
K91	1.9E+02	1.1E+02		
H92	2.6E+02	1.2E+02	2.2E+02	1.7E+02
G96	5.8E+01	3.9E+01	1.0E+02	7.9E+01
S99	5.9E+02	2.2E+02	5.2E+02	3.3E+02
N102	9.6E+01	2.2E+01	1.5E+02	5.5E+01
G109	3.2E+02	1.4E+02		
S110	1.0E+03	2.2E+02		
Q111	5.8E+02	1.4E+02	1.5E+02	3.0E+01
F113	2.8E+02	8.3E+01	1.4E+02	3.7E+01
I114	1.4E+02	8.5E+01		
C115	1.1E+03	8.6E+02	1.2E+02	9.9E+01
A117	1.7E+02	2.5E+01	1.5E+02	3.1E+01
T119	7.0E+02	6.1E+02		
E120	6.2E+02	1.4E+02	4.5E+02	2.0E+02
W121	2.1E+02	1.8E+02		
L122	7.5E+01	2.7E+01	8.7E+01	5.2E+01
D123	1.7E+02	2.5E+01	1.5E+02	3.1E+01
G124	4.1E+02	4.0E+02	2.3E+02	2.2E+02
V127	1.0E+03	1.8E+02	2.9E+02	1.7E+02
K131			1.1E+02	7.0E+01
V132	1.2E+02	5.6E+01	1.5E+02	1.5E+02
R144	6.5E+02	4.9E+02	2.1E+02	1.5E+02
T152			1.2E+02	5.7E+01
T157	9.9E+01	3.6E+01	1.2E+02	2.6E+01
I158			8.8E+01	8.1E+01

Table S2. Kinetic parameters used for theoretical kinetic analysis.

	–PPIA	Wild-type PPIA	K76E
Pep$_{trans}$ → Pep$_{cis}$ (10^{-3} s^{-1})	0.81[a]	0.81[a]	0.81[a]
Pep$_{cis}$ → Pep$_{trans}$ (10^{-3} s^{-1})	6.8[a]	6.8[a]	6.8[a]
Pep$_{trans}$ + PPIA → Pep$_{trans}$·PPIA (10^{6} s^{-1} M^{-1})		3.2[b]	3.2[b]
Pep$_{trans}$·PPIA → Pep$_{trans}$ + PPIA (s^{-1})		550[b]	550[b]
Pep$_{trans}$·PPIA → Pep$_{cis}$·PPIA (s^{-1})		1660[b]	150[c]
Pep$_{cis}$·PPIA → Pep$_{trans}$·PPIA (s^{-1})		1070[b]	100[c]
Pep$_{cis}$ + PPIA → Pep$_{cis}$·PPIA (10^{6} s^{-1} M^{-1})		10[b]	10[b]
Pep$_{cis}$·PPIA → Pep$_{cis}$ + PPIA (s^{-1})		170[b]	170[b]
Pep$_{cis}$ → Native (s^{-1})	0.1[d]	0.1[d]	0.1[d]

[a] Ref.[12]
[b] Ref.[10]
[c] Value to approximately 1/10 of the wild-type based on the results of the CPMG experiment in this study.
[d] Value optimized to correspond roughly to the timescale of the RNase T1 refolding experiment (Figure S9).

References

(1) Babu, M., Favretto, F., de Opakua, A. I., Rankovic, M., Becker, S., and Zweckstetter, M. (2021) Proline/arginine dipeptide repeat polymers derail protein folding in amyotrophic lateral sclerosis. *Nat. Commun. 12*, 1–7.

(2) Delaglio, F., Grzesiek, S., Vuister, G. W., Zhu, G., Pfeifer, J., and Bax, A. (1995) NMRPipe: A multidimensional spectral processing system based on UNIX pipes. *J. Biomol. NMR 6*, 277–293.

(3) Lee, W., Rahimi, M., Lee, Y., and Chiu, A. (2021) POKY: a software suite for multidimensional NMR and 3D structure calculation of biomolecules. *Bioinformatics 37*, 3041–3042.

(4) Fielding, L. (2007) NMR methods for the determination of protein–ligand dissociation constants. *Prog. Nucl. Magn. Reson. Spectrosc. 51*, 219–242.

(5) Loria, J. P., Rance, M., and Palmer, A. G., 3rd. (1999) A TROSY CPMG sequence for characterizing chemical exchange in large proteins. *J. Biomol. NMR 15*, 151–155.

(6) Sugase, K., Konuma, T., Lansing, J. C., and Wright, P. E. (2013) Fast and accurate fitting of relaxation dispersion data using the flexible software package GLOVE. *J. Biomol. NMR 56*, 275–283.

(7) Luz, Z., and Meiboom, S. (1963) Nuclear magnetic resonance study of the protolysis of trimethylammonium ion in aqueous solution—order of the reaction with respect to solvent. *J. Chem. Phys. 39*, 366–370.

(8) Wang, J., Jain, A., McDonald, L. R., Gambogi, C., Lee, A. L., and Dokholyan, N. V. (2020) Mapping allosteric communications within individual proteins. *Nat. Commun. 11*, 3862.

(9) Johnson, K. A., Simpson, Z. B., and Blom, T. (2009) Global kinetic explorer: a new computer program for dynamic simulation and fitting of kinetic data. *Anal. Biochem. 387*, 20–29.

(10) Holliday, M. J., Armstrong, G. S., and Eisenmesser, E. Z. (2015) Determination of the full catalytic cycle among multiple cyclophilin family members and limitations on the application of CPMG-RD in reversible catalytic systems. *Biochemistry 54*, 5815–5827.

(11) Holliday, M. J., Camilloni, C., Armstrong, G. S., Vendruscolo, M., and Eisenmesser, E. Z. (2017) Networks of dynamic allostery regulate enzyme function. *Structure 25*, 276–286.

(12) Schiene, C., Reimer, U., Schutkowski, M., and Fischer, G. (1998) Mapping the stereospecificity of peptidyl prolyl cis/trans isomerases. *FEBS Lett. 432*, 202–206.
